# Improving Door-to-balloon Time by Decreasing Door-to-ECG time for Walk-in STEMI Patients

**DOI:** 10.5811/westjem.2014.10.23277

**Published:** 2014-12-09

**Authors:** Christopher J. Coyne, Nicholas Testa, Shoma Desai, Joy Lagrone, Roger Chang, Ling Zheng, Hyung Kim

**Affiliations:** * Los Angeles County/University of Southern California, Department of Emergency Medicine, Los Angeles, California; † Los Angeles County/University of Southern California, Los Angeles, California

## Abstract

**Introduction:**

The American Heart Association/American College of Cardiology guidelines recommend rapid door-to-electrocardiography (ECG) times for patients with ST-segment elevation myocardial infarction (STEMI). Previous quality improvement research at our institution revealed that we were not meeting this benchmark for walk-in STEMI patients. The objective is to investigate whether simple, directed changes in the emergency department (ED) triage process for potential cardiac patients could decrease door-to-ECG times and secondarily door-to-balloon times.

**Methods:**

We conducted an interventional study at a large, urban, public teaching hospital from April 2010 to June 2012. All patients who walked into the ED with a confirmed STEMI were enrolled in the study. The primary intervention involved creating a chief complaint-based “cardiac triage” designation that streamlined the evaluation of potential cardiac patients. A secondary intervention involved moving our ECG technician and ECG station to our initial triage area. The primary outcome measure was door-to-ECG time and the secondary outcome measure was door-to-balloon time.

**Results:**

We enrolled 91 walk-in STEMI patients prior to the intervention period and 141 patients after the invention. We observed statistically significant reductions in door-to-ECG time (43±93 to 30±72 minutes, median 23 to 14 minutes p<0.01), ECG-to-activation time (87±134 to 52±82 minutes, median 43 to 31 minutes p<0.01), and door-to-balloon time (134±146 to 84±40 minutes, median 85 -75 minutes p=0.03).

**Conclusion:**

By creating a chief complaint-based cardiac triage protocol and by streamlining ECG completion, walk-in STEMI patients are systematically processed through the ED. This is not only associated with a decrease in door-to-balloon time, but also a decrease in the variability of the time sensitive intervals of door-to-ECG and ECG-to-balloon time.

## INTRODUCTION

### Background

Expedited treatment of ST-segment elevation myocardial infarction (STEMI) with percutaneous coronary intervention (PCI) has been shown to improve outcomes.^1^–^4^ Multiple strategies may be employed to improve the efficiency with which STEMI patients are evaluated and treated once they arrive to the emergency department (ED). These include (but are not limited to) early electrocardiography (ECG), prompt ECG interpretation, early catheterization lab activation, an expedited response to activation, and rapid reperfusion.

Previous quality improvement research at our facility was designed to investigate these intervals and to identify areas of deficiency. It was noted through this investigation that there was a significant difference between door-to-ECG times in those patients that arrived to the ED via ambulance versus those patients who walked in.^5^ Previous American Heart Association/American College of Cardiology (AHA/ACC) guidelines have recommended rapid door-to-ECG times (less than 10 minutes) for suspected STEMI patients, and studies have shown that there is an increased risk of poor outcomes if this specific metric is delayed.^6^,^7^ Although our institution met this goal for patients arriving by ambulance (median eight minutes), the door-to-ECG time for walk-in patients (median 23 minutes) was significantly delayed.

Previous data have shown that simple, directed changes to ED triage can significantly affect door-to-ECG time and secondarily door-to-balloon time.^8^ These studies have generally focused on smaller, low-to-moderate volume EDs, while our study focuses on a large volume, crowded ED. Patients with chest pain arriving to our ED via ambulance are quickly triaged and an ECG is rapidly obtained. Prior to this study, however, ambulatory patients with chest pain underwent a more lengthy, delayed triage system prior to ECG. In an effort to improve our walk-in door-to-ECG times, our department designed a new pathway for evaluating and treating patients with chief complaints consistent with acute coronary syndrome (ACS).

### Goals of this study

This study was designed to investigate whether simple, directed changes in the initial evaluation of cardiac patients could significantly affect our door-to-ECG times and secondarily our door-to-balloon times for patients who walked into the ED.

## METHODS

### Study Design and Setting

We conducted this interventional study at a large, urban, public teaching hospital with an annual census of approximately 175,000 patients. It was conducted from April 2010 to June 2012. We included in this study, all walk-in patients with a confirmed ST elevation myocardial infarction. The university institutional review board approved this study.

### Pre-Intervention Protocol

From April 2010 through March 2011 we tracked the door-to-ECG times for all walk-in STEMI patients who presented to the ED. The initial intake process during this period consisted primarily of a nurse who designated an urgent or emergent triage category for each patient depending on the severity of the individual’s general appearance and chief complaint. Emergent category patients, including all patients with chest pain, were placed in line for an expedited assessment. During this process, the assessment nurse or mid-level provider would receive a brief history and obtain appropriate labs and basic diagnostic studies as part of an early medical screening exam. If an ECG was indicated, the patient was transferred to a separate designated area, where an ECG technician performed this study and requested a physician interpretation. If a STEMI was identified at this time, the cardiology team and catheterization laboratory were alerted. If the physician did not detect STEMI on ECG, the patient was returned to the triage area to await further provider evaluation. Urgent category patients were placed in line for a delayed triage assessment.

### Intervention

From April 2011 through June 2011, we implemented new changes to the ED intake system in an attempt to decrease door-to-ECG times for STEMI patients. These changes included creating a Cardiac Triage designation that was assigned by the ED intake nurse upon arrival based on chief complaint. The Cardiac Triage designation (an addition to our urgent and emergent designations) prioritized the patients in an electronic patient tracking system. Patients were designated as cardiac triage if they had either chest pain, shortness of breath, or if they were thought to have other anginal equivalents based on the triage provider’s interpretation. In addition, the ECG technician and machine were moved to the area where the initial triage assessment took place. This was done not only to eliminate the technician transit time, but also to create an increased sense of urgency with regards to the ECG.

### Post-Intervention Protocol

From July 2011 through June 2012, we again tracked door-to-ECG times for all STEMI patients. The intake nurse designated all patients as urgent, emergent or cardiac, depending on their chief complaints. Urgent and emergent patients were triaged as noted above. Cardiac patients either receive an immediate ECG in the triage area itself, or if there were mid-level providers available, they received the initial medical screening exam and ECG simultaneously. The ECG was then brought to a physician for interpretation and if indicated, a “code STEMI” was called to notify cardiology and activate the cath lab.

### Outcomes and Data Collection

The primary endpoint for this study is the door-to-ECG time. The secondary end points include activation time (ECG to “code STEMI” activation), response time (“code STEMI” activation to arrival of cath team), cath lab arrival time (response to patient arrival to cath lab), attending arrival time (patient in cath lab to attending arrival to cath lab), balloon time (attending in cath lab to balloon time), and overall door-to-balloon time. In addition, we also prospectively collected the demographic characteristics of the walk-in patients. We entered and stored all data in an ACCESS database. All data captured were double-checked by a dedicated research coordinator for accuracy. We performed data quality control on a quarterly basis to identify outliers and fallout cases.

### Statistical Analysis

Mean and median time intervals are reported for each discrete step from ED arrival-to-balloon time. We examined distribution of each variable to determine the appropriate statistical approach used in the analysis. The Wilcoxon Rank-Sum Test was used to compare the mean times between the two groups for each step. We used the Chi-square test or the 2-sided Fisher’s exact tests to compare proportions between two groups. A p-value less than or equal to 0.05 was considered statistically significant. We performed all the statistical analyses with the statistical software SAS v9.1.

## RESULTS

### Characteristics of Study Subjects

The analysis is based on a dataset of 232 walk-in STEMI patients before and after the intervention period (April 2011 through June 2011) at the Los Angeles County+University of Southern California Medical Center. Ninety-one patients were encountered from April 2010 through March 2011 prior to the intervention, while 141 patients were encountered from July 2011 through June 2012 after the intervention. The data collected during the three-month intervention period were piloted but not included in the analysis.

The demographic characteristics for walk-in STEMI patients before and after the intervention are presented in Table 1. Of note, approximately 10% of overall walk-in patients were placed in the cardiac triage category. We found no statistically significant differences in the median age (p=0.1), gender distribution (p=0.7), ethnicity distribution (p=0.1), or prevalence of chest pain as initial complaint (p=0.1) between the two groups.

### Main Results

The primary and secondary endpoints are compared between the two groups in Table 2. We observed statistically significant reductions in both mean and SD for the following intervals: (1) door-to-ECG time (43±93 to 30±72 minutes, median 23 to 14 minutes, p<0.01), (2) ECG-to-activation time (87±134 to 52±82 minutes, median 43 to 31 minutes p<0.01), and (3) door-to-balloon time (134±146 to 84±40 minutes, median 85 - 75 p=0.03) after the intervention period. Additionally, we observed decreased standard deviation in the time intervals for door-to-ECG, ECG-to-activation, and door-to-balloon after the intervention.

## DISCUSSION

Novel process changes in our ED were associated with a significant reduction in door-to-ECG time, ECG-to-catheterization lab activation time, and door-to-balloon time. Additionally, by streamlining the process for cardiac triage, ECG performance and ECG interpretation, we observed an associated decrease in the variability of these time intervals (Table 2). Much has been published on the topic of quality improvement measures to decrease door-to-balloon times. In 2006, Bradley et al.^9^ found that six strategies were associated with faster door-to-balloon times. These included having a single call to a page operator to activate the cath lab, having the ED activate the cath lab, pre-hospital cath lab activation, expecting cath lab staff to arrive within 20 minutes after being paged, having an attending cardiologist always on site and having staff in the ED and cath lab use real-time data feedback. The Door to Balloon Alliance subsequently added two additional strategies, including a team-based approach to STEMI and the involvement of senior hospital leadership in the STEMI action plan.^10^,^11^ Although these quality improvement strategies are generally efficacious, supported by interventional studies, they all focus specifically on the period after the diagnosis of STEMI is made.^12^,^13^ This may not be an issue for those patients arriving to the ED by ambulance, where virtually all patients with chest pain are seen immediately. The population of patients walking into the emergency department with chest pain, however, may encounter a significant delay in their diagnosis of STEMI and subsequent treatment, despite having the Bradley et al. best practice measures in place.

Expediting the diagnosis of STEMI and ED cath lab activation (Activation time) is paramount in the effort to decrease door-to-balloon times. Although pre-hospital diagnosis of STEMI has been shown to decrease door-to-activation times for patients arriving via ambulance, there are no best practice measures that address the door-to-activation time for walk-in patients.^14^ This is despite recent data, which demonstrates that door-to-activation time remains the key determinant of door-to-balloon times.^15^ The importance of door-to-activation time on door-to-balloon time was apparent in our own institution, where we noted a significant delay in door-to-balloon times for our ambulatory STEMI patients. This was primarily driven by our lengthy walk-in door-to-activation times. We successfully eliminated this delay by changing our initial triage system for cardiac patients and by moving our electrocardiogram (EKG) technician to the initial triage area.

Although there is a relative lack of research on door-toactivation time, the research that does exist has demonstrated the critical importance of appropriate ED triage. In a study on ED triage of patients with acute myocardial infarction (AMI) in 2011, Atzema et al.^8^ found that one-third of all AMI patients were given a low triage score, which was associated with a significant delay in door-to-ECG and door-to-balloon times. Additionally, they found that 26% of STEMI patients were placed in this low-priority triage group, which was associated with increased length of stay and mortality. In an effort to avoid these issues in our ED triage system, we created a new “cardiac” triage category to prioritize patients with chief complaints consistent with acute myocardial infarction. This chief complaint-based, rapid triage for potential AMI patients has been shown to significantly decrease time to definitive therapy.^16^ Our new triage system allows cardiac patients to forego the regular triage system and to receive a rapid ECG.

To further expedite our time to ECG, we moved our ECG technician to our preliminary triage area. This allowed for an EKG to rapidly take place, immediately after the patient stated their chief complaint. This is critically important, given that increased time to ECG has been associated with increased mortality in patients with STEMI.^17^ Previously, these studies were delayed at our institution, due to the physical distance between the EKG technician and the patient. The location change also introduced a sense of urgency, given that these chest pain patients were placed immediately in front of the EKG tech station. Together, these ED interventions were not only associated with a decrease in our door-to- balloon times, but also a decrease in the variability of the critical intervals of door-to-ECG and ECG-to-cath lab activation.

## LIMITATIONS

One limitation of this study was that we changed our variables simultaneously and therefore cannot state whether it was our development of a “cardiac” designation at triage, or the movement of the ECG technician that decreased our door-to-ECG time. We propose that both of these interventions likely contributed to our improved intervals and that each change would be efficacious individually at other institutions. A second limitation of our study was that it was done at a single center and therefore may lack external validity. We propose that similar triage protocols are warranted and feasible in all EDs, especially those in which high patient volumes may delay the expedited treatment of potential STEMI patients. Third, although we significantly decreased our door-to-ECG times, we did not meet recommended AHA/ACC guideline for door-to-ECG time (14 minutes vs 10 minute benchmark). Regardless, our interventions were associated with not only a decrease in door-to-ECG times, but also door-to-activations times, which dropped our door-to-balloon times below the 90-minute benchmark. Furthermore, with continued efforts within our ED beyond the study period, these intervals anecdotally continued to decrease, though follow-up studies are necessary to confirm this phenomenon. Finally, it is possible that there are other potential confounders that affected our final interval times. One could argue that the staff knowledge of these interval measurements may have brought down our post-intervention times. That being said, these intervals were tracked long before our intervention period, and there had already been a push for decreased door-to-balloon time prior to the pre-intervention time period.

## CONCLUSION

Through process changes in the emergency department management of cardiac patients, one can observe an associated decrease in door-to-balloon times for walk-in STEMI patients. With a chief complaint-based “cardiac” triage designation, patients are rapidly triaged, an immediate ECG is obtained, and the catheterization lab is expeditiously activated. With a separate cardiac triage protocol, these patients are systematically processed through the ED, which is associated with decreases in door-to-balloon times, as well as decreased variability in the time sensitive intervals of door-to-ECG and ECG-to-balloon time. Finally, by maintaining a designated ECG technician in the ED triage area, unnecessary delays in the diagnosis and treatment of STEMI patients are eliminated.

## Figures and Tables

**Table 1:** Demographic characteristics of STEMI patients pre- and post-intervention.

Characteristic	Pre-intervention (N=91)	Post-intervention (N=141)	p-value†‡
Age* (Year)	57.1 ± 11	55 ± 11	0.1
Gender % (n)			
Male	70.3% (64)	72.3% (102)	0.7
Female	29.7% (27)	27.7% (39)	
Ethnicity % (n)			
White	9.9% (9)	8.5% (12)	0.1
African American	5.5% (5)	12.8% (18)	
Asian	18.7% (17)	12.1% (17)	
Latino	62.6% (57)	64.5% (91)	
Other	3.3% (3)	2.1% (3)	
Latino % (n)			
Yes	62.6% (57)	64.5% (91)	0.8
No	37.4% (34)	35.5% (50)	
Chest pain as initial complaint, % (n)	73.3% (66)	83.7% (118)	0.1

*STEMI,* ST segment elevation myocardial infarction

* Mean ± SD.

† Obtained from chi-square test except from Wilcoxon rank-sum test for age.

‡ P value for ethnicity applies to all subgroups.

**Table 2:** Comparison of time intervals in STEMI patients pre- and post-intervention.

	Pre-intervention		Post-intervention		
		
Time intervals	N	Mean ± SD (minutes) Median (Min,Max) (minutes)	N	Mean ± SD (minutes) Median (Min,Max) (minutes)	p-value*
Door-to-ECG time	91	43.1 ± 93.1 23 (0, 636)	141	30.3 ± 71.6 14 (2, 507)	<0.01
ECG-to-activation time	90	87.1 ± 133.8 42.5 (3, 724)	131	51.6 ± 81.6 31 (5, 539)	<0.01
Activation to response time	91	5 ± 0.2 5 (4, 6)	126	4.9 ± 0.5 5 (0, 6)	0.3
Response to patient in lab time	63	14.4 ± 5 14 (2, 30)	75	16.1 ± 15.2 13 (1, 127)	0.3
Patient in lab to attending time	62	11.2 ± 7.4 9.5 (1, 26)	73	9 ± 5.7 7 (1, 24)	0.2
Attending to balloon time	43	22.5 ± 10.9 21 (9, 71)	57	22 ± 12.1 19 (10, 79)	0.4
Door-to-balloon time	43	134.3 ± 146.3 85 (44, 709)	57	83.9 ± 39.8 75 (44, 243)	<0.05

*STEMI,* ST segment elevation myocardial infarction; *ECG,* electrocardiogram

* Obtained from Wilcoxon rank-sum test.
